# Multiple cartilaginous exostosis in a feline: Case report

**DOI:** 10.29374/2527-2179.bjvm004526

**Published:** 2026-11-05

**Authors:** Adriano Sílvio

**Affiliations:** 1 Departamento de Medicina Veterinária, Faculdade do Futuro, Manhuaçu, MG, Brazil

**Keywords:** osteocartilaginous exostosis, feline, radiograph, exostose osteocartilaginosa, felino, radiografia

## Abstract

Multiple cartilaginous exostoses (MCE), or osteochondromatosis, is a benign neoplasm characterized by the presence of nodules on the bone surface. In felines, the condition may be associated with feline leukemia virus (FeLV) infection and genetic mutations. This study reports the case of a 2-year-old cat diagnosed with MCE. The animal presented with cachexia, hyporexia, dehydration, and increased bone volume in multiple anatomical regions. Radiographic examination revealed proliferative sclerotic lesions, and histopathology confirmed osteochondrogenic proliferation consistent with MCE. Initial treatment included doxycycline and prednisolone. After 30 days, the cat showed clinical improvement; however, the osseous proliferations continued to increase in size. Subsequent radiographic evaluation demonstrated marked lesion progression, accompanied by worsening clinical signs. Due to the extent of the lesions and the unfavorable clinical condition, euthanasia was elected. This report highlights the clinical and imaging features of MCE in a young FeLV-positive cat and emphasizes the importance of early diagnosis and imaging follow-up in the management of affected patients.

## Resumo

A exostose cartilaginosa múltipla (ECM), ou osteocondromatose, é uma neoplasia benigna caracterizada pela presença de nódulos na superfície óssea. Em felinos, a condição pode estar associada à infecção pelo vírus da leucemia felina (FeLV) e a mutações genéticas. Este estudo relata o caso de uma gata de 2 anos diagnosticada com ECM. O animal apresentava caquexia, hiporexia, desidratação e aumento de volume ósseo em múltiplas regiões anatômicas. O exame radiográfico revelou lesões proliferativas escleróticas, e a histopatologia confirmou proliferação osteocondrogênica compatível com ECM. O tratamento inicial incluiu doxiciclina e prednisolona. Após 30 dias, a felina apresentou melhora clínica; entretanto, as proliferações ósseas continuaram a aumentar de tamanho. A avaliação radiográfica subsequente demonstrou progressão acentuada das lesões, acompanhada pelo agravamento dos sinais clínicos. Devido à extensão das lesões e à condição clínica desfavorável, optou-se pela eutanásia. Este relato destaca as características clínicas e de imagem da ECM em uma gata jovem positiva para FeLV e enfatiza a importância do diagnóstico precoce e do acompanhamento por métodos de imagem no manejo de pacientes acometidos.

## Introduction

Multiple cartilaginous exostosis (MCE) is a benign neoplasm characterized by the presence of a single nodule (osteochondroma) or multiple nodules (osteochondromatosis) on the surface of cartilage-covered bones. In felines, its occurrence may be associated with the feline leukemia virus (FeLV) (Fujii et al., 2022). There are reports of cats infected with FeLV and diagnosed with MCE in which C-type retroviral particles similar to FeLV and feline sarcoma virus were observed by electron microscopy in the cartilaginous caps (Gómez et al., 2023).

Among the tumors diagnosed in this species, fewer than 5% are primary bone neoplasms, and MCE is included among them (Fujii et al., 2022). In addition to the correlation with FeLV, the disease has been associated with mutations in the exostosin glycosyltransferase 1 and 2 genes (EXT1 and EXT2) as a result of an autosomal dominant disorder (Gómez et al., 2023).

MCE typically appears only until the closure of the epiphyseal plate. However, in felines, osteocartilaginous growth may be detected even after skeletal maturity, indicating causes other than autosomal genetic disorders (Thompson, 2017).

The average age of onset of the lesions is approximately 2 to 3 years, with no known sex predisposition. The most commonly described anatomical locations include the vertebral column, long bones, head, ribs, and pubis. The clinical signs developed by the animal depend on the location of the lesions and the mechanical impact on surrounding structures (Nolff et al., 2014).

Feline MCE tumors show progressive development and may significantly deteriorate the patients’ quality of life. In addition, malignant transformation to osteosarcoma or chondrosarcoma may occur (Szilasi et al., 2022). The main objective of this study was to describe the case of a 2-year-old, 2.5-kg, mixed-breed female cat diagnosed with MCE based on clinical signs, physical examination, laboratory tests, serial radiographs, and histopathology.

## Case report

A 2-year-old, 2.5-kg, mixed-breed female cat was presented with cachexia, pale mucous membranes, hyporexia, prostration, 8% dehydration, and discrete, circumscribed swellings in the proximal regions of the mandible and maxilla (Figure 1A and E), left mandibular bone, right occipital condyle (Figure 1B and F), left scapula (Figure 1C and G), pelvis (Figure 1D and H), and an extraskeletal mass located dorsally to the fourth and fifth cervical vertebrae (Figure 1G). Radiographic images of these regions were obtained, revealing amorphous, proliferative, and sclerotic structures. A complete blood count and testing for feline leukemia virus (FeLV) were requested. In addition, an incision was made in the lesion near the mandibular symphysis to remove a 0.5 cm fragment for histopathological examination.

**Figure 1:**
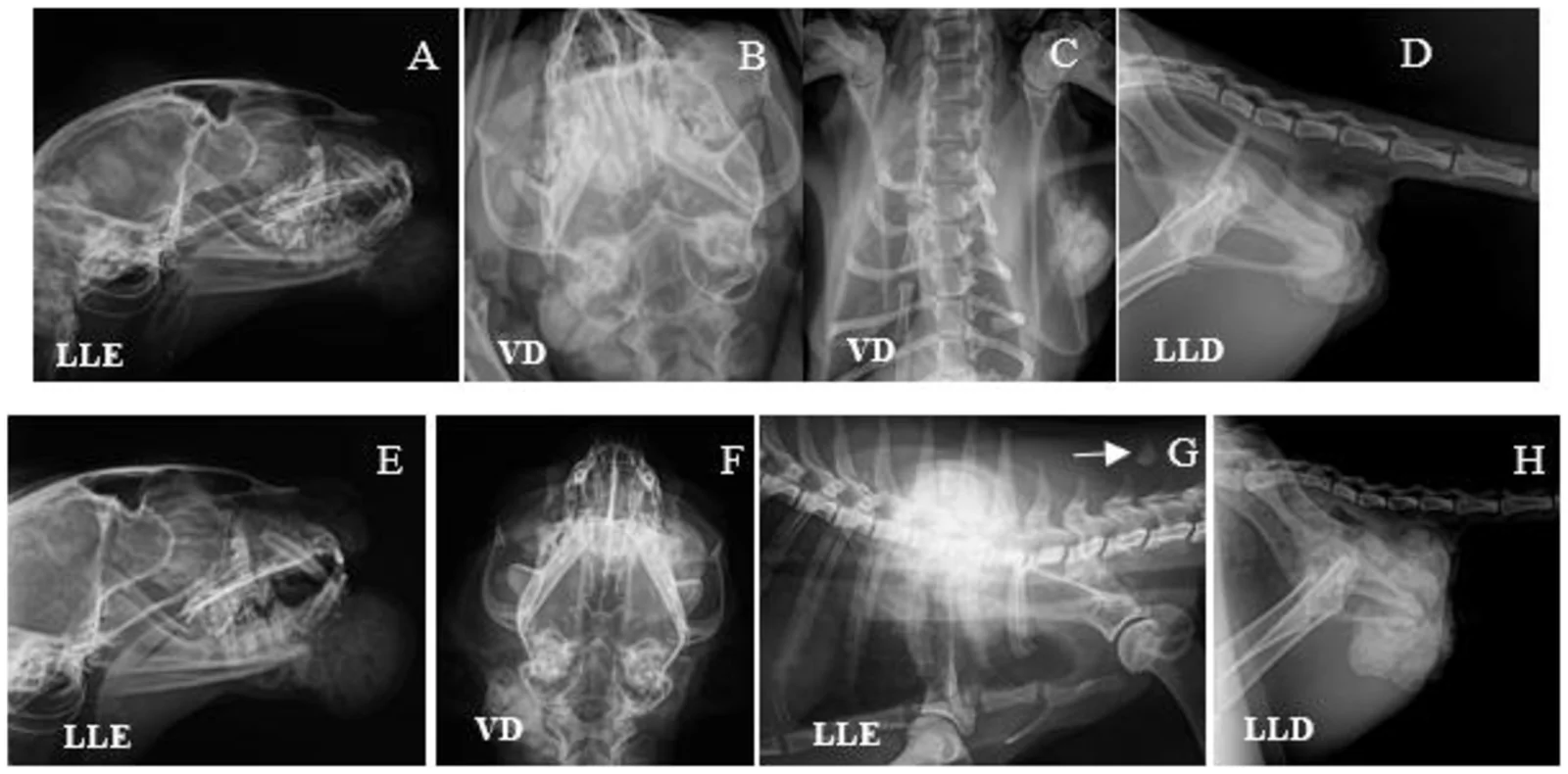
Radiographs obtained at two different time points from a 2-year-old, mixed-breed female cat diagnosed with multiple cartilaginous exostosis. In the mandibular region, an irregular, amorphous, sclerotic bone proliferation is observed in the rostral portion of the mandible, near the incisor teeth, causing lysis of the adjacent mandibular bone with destruction of the cortical bone (A/E). Amorphous, irregular, proliferative, and sclerotic structures are also present in the region of the left scapula (C/G), right occipital condyle (B/F), ischial region of the pelvis (D/H), and in soft tissues dorsal to the fourth cervical vertebra (G – white arrow). Legend: LLE: left laterolateral; VD: ventrodorsal; LLD: right laterolateral.

Laboratory tests demonstrated anemia (hematocrit of 22%), leukopenia (2,800 ×10^3^/µL), and hyperproteinemia (8.2 g/dL). The FeLV test result was positive. Histopathology revealed osteochondrogenic proliferation characterized by a hyaline cartilage surrounded and delimited by bony trabeculae, a finding consistent with multiple cartilaginous exostosis (MCE).

The animal was treated with doxycycline (10 mg/kg, SID) for 15 days, prednisolone (1 mg/kg, BID) for 10 days, and Hemolitan (0.3 mL, BID) for 30 days. At the follow-up visit after 30 days, the owner reported improved appetite and weight gain. However, the bony neoformations showed progressive growth. Fourteen days after the second consultation, the animal returned to the clinic presenting anorexia, dysphagia, dyschezia, and progressive weight loss. The cat was referred for new radiographs, which demonstrated a significant increase in the polyostotic neoformations, impairing locomotion and the ingestion of food and water. Due to the impossibility of surgically resecting all lesions and the unfavorable prognosis, the animal was euthanized at the owner’s request.

The interval between Figures 1A, B, C, and D and Figures 1E, F, G, and H was 44 days. A significant increase in bone proliferation was observed, leading to impaired vital functions of the animal, such as obtaining food, ingesting liquids, walking, and defecating.

## Discussion

The present report describes a young FeLV-positive cat with rapidly progressive multiple cartilaginous exostoses (MCE), characterized by extensive polyostotic proliferations and marked clinical deterioration. The animal age is consistent with studies describing MCE predominantly in young adult cats (Szilasi et al., 2022), although Nolff et al. (2014) reported a higher mean age at diagnosis. This reinforces that MCE should be considered a differential diagnosis in young felines presenting with proliferative bone lesions, regardless of sex, since no sex predisposition has been consistently demonstrated.

The distribution of lesions observed in this case partially overlaps with the anatomical sites most frequently reported for feline MCE, including the vertebral column, long bones, head, ribs, and pubis (Nolff et al., 2014). In addition, the detection of an extraskeletal osteochondroma dorsal to the cervical vertebrae resembles the unusual extraskeletal presentation described by Rosa & Kirberger (2012) in a Burmese cat. Such findings highlight the disease’s morphological variability and the importance of imaging evaluation beyond the appendicular skeleton.

The hematological abnormalities observed in the present case were more likely related to FeLV infection than to osteochondromatosis itself. Progressive FeLV infection is commonly associated with anemia, leukopenia, and other bone marrow-related disorders (Advisory Board on Cat Diseases, 2021), whereas MCE is generally not associated with significant hematologic alterations (Gómez et al., 2023; Reis et al., 2017). Therefore, the laboratory findings support the coexistence of FeLV infection rather than a direct systemic effect of the osteocartilaginous lesions.

Histopathology confirmed the diagnosis of MCE by demonstrating osteochondrogenic proliferation with hyaline cartilage surrounded by bony trabeculae, a pattern consistent with previous descriptions of feline osteochondromatosis (Reis et al., 2017; Winter et al., 2017). Importantly, no histological evidence of malignancy was identified in the sampled tissue, reinforcing the benign nature of the lesion at the time of diagnosis.

Radiographically, the disease exhibited an aggressive pattern, showing extensive sclerotic proliferations, cortical lysis, and rapid progression over just 44 days. Similar radiographic abnormalities have been described by Szilasi et al. (2022), who reported poorly defined calcified lesions deforming thoracic structures and invading body cavities. In contrast, Nolff et al. (2014) and Gómez et al. (2023) described more circumscribed proliferations without significant bone destruction, compatible with a more indolent presentation. Thus, the present case appears to represent a clinically severe form of MCE, in which the imaging findings suggest a more aggressive biological behavior, although malignant transformation could not be confirmed histologically.

The relationship between FeLV infection and MCE remains controversial. Earlier studies suggested an association between FeLV and osteochondromatosis (Thompson, 2017), and retroviral particles resembling FeLV have been identified in cartilaginous caps in some cases. Nevertheless, the report by Gómez et al. (2023) of MCE in an FeLV-negative elderly cat demonstrates that the disease can occur independently of FeLV infection. Therefore, while the coexistence of FeLV and MCE in the present case is noteworthy, it does not establish a causal relationship. The current evidence is more consistent with a possible association than with a proven etiological role of FeLV in osteochondrogenic proliferation.

From a clinical standpoint, this case illustrates the poor prognosis associated with diffuse and rapidly progressive MCE. Although surgical excision may be attempted in selected cases (Nolff et al., 2014), the multiplicity and distribution of lesions in the present patient precluded curative resection. In this context, advanced imaging modalities such as computed tomography and magnetic resonance imaging may provide better lesion characterization, improve assessment of surgical feasibility, and assist in prognostic evaluation (Rosa & Kirberger, 2012).

## Conclusion

The coexistence of FeLV infection and MCE in this case supports the need for further investigation into the potential relationship between these conditions. The radiographic alterations observed, characterized by extensive sclerotic proliferations and areas of cortical lysis, suggested a more aggressive biological behavior and rapid progression, consistent with a guarded prognosis. Considering the absence of curative therapies and the progression observed in the present case, the importance of early diagnosis and the use of advanced imaging methods for clinical monitoring and therapeutic planning in these patients should be emphasized.
